# Visual Representations of Sexual Violence in Online News Outlets

**DOI:** 10.3389/fpsyg.2017.00774

**Published:** 2017-05-16

**Authors:** Sandra Schwark

**Affiliations:** Department of Psychology, Bielefeld UniversityBielefeld, Germany

**Keywords:** sexual violence, gender, media, qualitative analysis, rape myths

## Abstract

To study visual representations of sexual violence, photographs accompanying German Internet news articles that appeared between January 2013 and March 2015 (*N* = 42) were subjected to thematic analysis. Two main themes, consisting of several sub-themes, emerged from the data. The first theme was “rape myths,” illustrating a stereotypical view of sexual violence. It consisted of three sub-themes: “beauty standards,” referring to the fact that all women in our sample fit western beauty standards, “physical violence,” as most images implied some form of physical violence, and finally “location,” suggesting that rape only happens in secluded outdoor areas. These findings suggest that the images from our sample perpetuate certain rape myths. The second theme was “portrayal of victimhood,” referring to the way victims of sexual violence were portrayed in photographs. The analysis of the sub-theme “passivity” showed that these portrayals fit a certain stereotype: the women were shown to be weak and helpless rather than individuals with agency and able to leave their status as a victim. Further sub-themes were “background,” “organization of space,” “camera perspective,” and “lighting.” We discuss these findings in relation to possibly reinforcing rape myths in society and as an issue in creating a biased perception of women who have experienced sexual violence.

## Introduction

Photographs have been part of news media since the mid 19th century. Often, a simple image can convey more meaning than a written or oral account. In this study, we aim to examine what kind of messages photographs accompanying articles about sexual violence against women in online news outlets convey to their viewers. In order to answer this question, we have conducted a qualitative thematic analysis of a set of photographs that were obtained from reports about sexual violence in German online news outlets. This analysis yielded the two main themes “rape myths” and “portrayal of victimhood,” each with at least one sub-theme. Overall, we were able to show that several different rape myths were present in the analyzed pictures, such as the misconception that sexual assault happens in outdoor locations, or that physical violence is always involved in these crimes. Furthermore, our data showed a one-sided portrayal of women who have experienced sexual violence. In our sample, these women were portrayed as passive victims, lacking agency and self-efficacy. We discuss the implications of these results regarding their impact on public perceptions on both sexual violence itself as well as its survivors.

Sexual violence against women is an on-going and widespread problem. A report by the United Nations Statistics Division (2015) found that one in three women worldwide has experienced physical or sexual violence at some point in their lives. While the prevalence of sexual violence is high, the conviction rate remains low. In Germany, for example, only 15% of suspects in reported rape cases were convicted in the year 2006 (Jehle, 2012). As many researchers have pointed out, one of the underlying causes of this discrepancy are rape myths (Lonsway and Fitzgerald, 1994; Bohner et al., 2009).

### Rape Myths

The concepts of rape myths and rape myth acceptance (RMA) were first introduced by Schwendinger and Schwendinger (1974), as well as Brownmiller (1975). Schwendinger and Schwendinger (1974) focus on the idea of the myth of the impossibility of rape, describing the misconception that rape can easily be avoided, for example, by the woman’s resistance. In her paper, Burt (1980, p. 217) picks up the concept and further describes it as “stereotypes and myths – defined as prejudicial, stereotyped, or false beliefs about rape, rape victims, and rapists – creating a climate hostile to rape victims.” In later research, Bohner et al. (2009, p. 19) identified “four general types of rape myth: beliefs that

– *blame the victim for their rape* (e.g. ‘women have an unconscious desire to be raped’, ‘women provoke rape through their appearance and behavior’);
– express a *disbelief in claims of rape* (e.g. ‘most charges of rape are unfounded’, ‘women tend to exaggerate how much rape affects them’);
– *exonerate the perpetrator* (e.g. ‘most rapists are over-sexed’, ‘rape happens when a man’s sex drive gets out of control’);
– allude that *only certain types of women are raped* (e.g. ‘a woman who dresses in skimpy clothes should not be surprised if a man tries to force her to have sex’, ‘usually it’s the women who do things like hang out in bars and sleep around that are raped’).”

Another example of a popular rape myth is that rape usually happens in secluded outdoor areas and that the perpetrator is a stranger, while statistics show clearly that in most cases, the victim knew the perpetrator before the attack (Bundeskriminalamt, 2015). Furthermore, the notion that women lie about rape and make false accusations can also be refuted by data, as only about 5% of rape accusations are false (Ferguson and Malouff, 2016).

The phenomenon of rape myths has been the subject of a number of investigations. One of the consistent findings is that men generally show higher RMA than women (Muir et al., 1996; Hammond et al., 2011). The term “rape myth acceptance” refers to the degree to which an individual believes in rape myths, such as the ones described above.

### Gender Stereotypes

The topic of sexual violence is surrounded by a variety of social beliefs on the subject. Apart from the issue of rape myths, which we have discussed above, there clearly is a gendered aspect to it. Women are commonly perceived to be victims of sexual violence, whereas men are seen as perpetrators (Howard, 1984). While crime statistics support these assumptions (Bundeskriminalamt, 2015), it is problematic to frame women as inherently vulnerable to violent crimes. This perception is rooted in stereotypical perceptions of women as a group. In social psychology, Lippmann (1922) famously coined the term “stereotype” as the “pictures in our heads” of social groups as well as individuals around us. Stangor and Lange (1994) describe stereotypes as a mental association between certain characteristics and a label of a social category. In the case discussed above, this could be the association between women and vulnerability.

Since stereotypes are so persistent in our society, it is likely that they are not completely inaccurate. Several studies have found that stereotypical perceptions of social groups have at least some truth to them (e.g., Judd et al., 1991; Swim, 1994). However, in most cases, this is due to the status and roles that different groups hold in society and not because of individual differences (e.g., Stangor, 2000). Women are perceived as “the weaker sex” not because they actually are, but because of their lower status compared to men in our society. This stereotype of women being weak and vulnerable heavily influences the way we, as a society, talk about female victimization. This of course also spills over to the criminal justice system and the way police as well as courts handle cases of sexual violence against women. Rape has one of the lowest conviction rates (e.g., Kelly et al., 2005) compared to other violent crimes. Research has investigated this thoroughly, and while part of the problem is within the system itself (usually there are no witnesses and it is a matter of the victim’s statement vs. the statement of the perpetrator), stereotypes and rape myths held by criminal justice personnel has also been identified to be an issue that might influence the conviction rate negatively (e.g., Krahé et al., 2008).

Findings like this show how stereotypical perceptions of both women and sexual violence against them can cause harm in ways one might not initially think of. Therefore, we aim to investigate what kind of possible stereotypes and myths are reproduced in news media imagery on the issue of violence against women.

### Representation of Sexual Violence in the Media

The way that sexual violence is framed in the media is of vital importance, as this portrayal often shapes public opinion on the matter (Soothill, 1991). In a recent study, O’Hara (2012) performed a lexical analysis that aimed to explore how news media portray sexual violence against women. Her results showed that a majority of the analyzed articles perpetuate rape myths. The perpetrator was often described as “a devious monster” (O’Hara, 2012, p. 256), whereas the victim was frequently blamed for the assault because of her behavior, for example, the way she dressed. This links back to the rape myth that perpetrators of sexual violence are often described as “crazy” and therefore not to blame for their actions (Bohner et al., 2009). These findings are supported by other studies. Rape myths have also been found in prime time television contents, perpetuating false ideas about sexual violence (Brinson, 1992; Cuklanz, 1996, 2000). Garland et al. (2015) found that mainstream comic books’ portrayals of sexual violence also reproduce rape myths. For example, the previously discussed myth that perpetrators act out of sexual desire and that they are mentally ill was supported in comic books.

Concerning the issue of rape myths in news media, Franiuk et al. (2008b) investigated both the prevalence and the effects of rape myths in the headlines of news articles, surrounding a high-profile case of alleged sexual assault, involving the basketball player Kobe Bryant. Their results showed that 10% of articles about the case had a rape myth-endorsing headline (e.g., “she is lying” or “she asked for it”). Furthermore, when they exposed participants to such headlines, men were more likely to endorse rape-supportive attitudes and less likely to think that the alleged perpetrator was guilty, compared to those exposed to headlines that did not endorse rape myths. In a similar study, Franiuk et al. (2008a) examined the prevalence of rape myths in newspaper articles surrounding the same high-profile case. They found that rape myths were present in more than one-third of the examined articles, the most common one being that the woman lied about the sexual assault.

Another issue regarding the way news media report on rape is the overrepresentation of cases involving false allegations. Gavey and Gow (2001) analyzed a number of media texts from New Zealand regarding their mentions of supposedly false rape allegations. They conclude that these kinds of articles perpetuate the myth that women tend to lie about sexual assault. Apart from the concern of perpetuating rape myths, there is another issue regarding news reports about rape that needs to be taken into consideration. In a recent study, Hollander and Rodgers (2014) investigated the portrayal of women’s resistance to sexual assault. Their findings showed that about two-thirds of articles did not mention resistance at all, and the remaining articles did so only to note that the attempt was in vain. The authors concluded that this kind of reporting reinforces the idea of women as passive victims without agency. This form of secondary victimization can have negative effects, as it takes away the ability to be a functioning actor, rather than a passive victim (Holstein and Miller, 1990).

Not only the content, but also the language used to write about sexual violence plays an important role in the general perception of the issue. Henley et al. (1995) found that participants generally showed more acceptance of violence against women after they had read a mock news report on rape that employed the passive voice, compared to one that used the active voice. Furthermore, male participants attributed less perpetrator responsibility and victim harm in the passive voice condition.

As discussed above, there has been a variety of research showing not only the misrepresentation of sexual violence and its victims in news media, but also the serious effects this bias may have on readers. Previous research indicated that rape myths are present in both the headlines and the articles themselves in the print news media. However, to the best of our knowledge, no study has yet examined the accompanying photographs that are presented alongside news articles.

### The Role of Photographs in News Media

The role that photographs play in news media (mostly newspapers) has been the subject of thorough scientific investigations. For example, Zillmann et al. (1999) exposed participants to news-magazine reports that featured a favorable portrayal of either side of the issue discussed in the article. They found that participants’ assessment of the presented issues was biased in favor of the implications given by the photographs. Furthermore, Knobloch et al. (2003) found that the incorporation of threatening images into an online news website led to a more frequent selection of associated articles, when participants were free to choose between articles featuring innocuous vs. threatening images. Reading times for these texts were also increased.

These findings were replicated by Sargent (2007), who investigated image effects on selective exposure to news stories. The results showed that the inclusion of threatening images accompanying news articles resulted in significantly longer self-exposure time to following text sections, even if these texts did not have an accompanying image. The authors argued that “the threatening image produced an affective reaction in readers that stimulated greater and more deliberate cognitive processing of following text that was devoid of an image” (Sargent, 2007, p. 720). These affective reactions to “sensational” pictures could be a way for journalists to appeal to readers’ curiosity and encourage reading of the accompanying articles.

### The Current Study

Because of the lack of existing research on this topic, we chose a qualitative study design. Therefore, our research question for this paper is “How are survivors of sexual violence portrayed in images posted along German online news outlet articles about sexual violence?”

We chose to limit the sampling to German websites to create boundaries on the cultural context. During the period of data collection for this study, a legal reform of the German law on sexual violence has been brought forward. There was no systematic difference in the kind of pictures we found in articles discussing the legislation change and those that did not. As we do not have any data from before the discussion about this legislation change started or from after it passed, we cannot make any statements about a possible impact it might have had on our data. Another reason for limiting the sampling to German websites was the manageability of the data, given the time frame for this research.

While research has not yet examined images in this context, we based the following hypotheses on previous, text-related findings.

H1: Pictures of women posted along articles in online news outlets perpetuate rape myths.
H2: The images portray survivors of sexual violence as passive.

## Materials and Methods

The sample was drawn by using Google as a search engine, using the search terms “rape,” “sexual violence,” and “sexual assault” (the equivalent terms in German: “Vergewaltigung,” “sexuelle Gewalt,” and “sexueller Übergriff”).

The pictures resulting from this search were part of articles published from January 1, 2013 to March 31, 2015. This period yielded sufficient data for analysis. Only articles in German were included and the search was narrowed down to URLs ending with .de. Furthermore, there were several exclusion criteria:

(1) Sources that did not fit the definition of a news outlet. News outlets included any kind of news media such as the online presence of newspapers, but also news-related content on websites run by organizations (e.g., the police or charities).
(2) Websites with audio or video content only.
(3) Articles that did not contain a picture accompanying the article.
(4) Articles discussing cases of under 18-year-olds either as victims or as perpetrators, since the theoretical framework for sexual violence against adolescents and children would go beyond the scope of this article.
(5) Articles behind a paywall that could not be accessed by the researcher.

In the initial online search, 63 articles were found. Twenty-one of those did not contain an image and were therefore excluded from the analysis, leaving a total of 42 pictures. The range of websites that did and did not contain images in their articles did not systematically differ in terms of type of source. Overall, the websites included local newspapers from different parts of Germany, national newspapers, websites from the German public-service television networks, as well as women’s magazines.

Several content types could be identified (see **Table 1**). Most of the specific cases that were reported on (*n* = 16) came from local newspapers, containing information about crimes that had been committed in the area. Reports on legislation change (*n* = 7) were primarily discussed in national newspapers, as well as television networks’ websites. There were several websites discussing measures of rape prevention (*n* = 6) as well as those discussing possible consequences for victims (*n* = 6) and offering advice in terms of, e.g., counseling. The category “other” (*n* = 7) refers to a variety of different content, for example, an interview with a convicted rapist.

**Table 1:** Contents of articles included in the analysis; *N* = 42.

Content	*n*
Specific case	16
Legislation change	7
Rape prevention	6
Consequences for victims	6
Other	7

All articles included in this study discussed male-on-female sexual violence, as opposed to female-on-male or same-sex violence. This was not a prerequisite set by the author, but a result of the search process. Several of the images came with an annotation, stating that they are generic pictures, hence indicating that the photographs are staged and do not portray actual events. This was especially the case for pictures showing any kind of assault. Therefore, it is safe to assume that the women as well as the alleged perpetrators portrayed in these photographs are professional photo models, rather than actual victims or perpetrators of sexual violence.

In order to approach the research question, visual thematic analysis was employed. Braun and Clarke (2006) provided a clear overview of the method, as well as giving instructions about its execution. However, their paper focused only on text-based thematic analysis. Gleeson (2011) has adapted a method of thematic analysis for visual data, called “polytextual thematic analysis.” The method of the research presented here was based largely on the aforementioned two papers. According to Braun and Clarke (2006), thematic analysis can be employed free from any epistemology. The approach used in the analysis is deductive, based on the specific research questions given earlier. As Gleeson (2011) points out, creating codes and themes from visual data requires taking these elements on a textual level. The identified themes are therefore to be seen as written descriptions of visual representation in the selected images.

Three independent coders were involved in the analysis. They viewed the pictures repeatedly, individually as well as in groups and different orders. During this process, we took notes and wrote down descriptions of the images. Next, relevant images were pulled together in the first attempts of identifying possible themes. These were noted down and subsequently revised several times. All of the researchers involved in this process were female, holders of a postgraduate degree in Psychology and trained in qualitative as well as quantitative research methods.

Once we had identified the final versions of the themes, we then looked at them in relation to each other to verify their distinctness. As a final step, we chose the themes for writing up that best fit to address the research questions. Three independent coders then coded each of the themes for each picture (present vs. absent). In order to assess inter-rater reliability, Krippendorff’s alpha was calculated for each of the sub-themes (see **Table 2**). For further information on calculating inter-rater reliability for qualitative data with this measure, see Hayes and Krippendorff (2007).

**Table 2:** Inter-rater reliability for sub-themes.

Theme 1: Rape myths		Theme 2: Portrayals	
Measure	Krippendorff’s alpha	Measure	Krippendorff’s alpha
Location	0.82	Passivity	0.83
Physical violence	0.91	Background	0.61
Beauty standards	0.82	Organization of space	0.70
		Camera perspective	0.62
		Lighting	0.73

*For all correlations and measures of association: *N* = 42.*

## Results and Discussion

**FIGURE 1:**
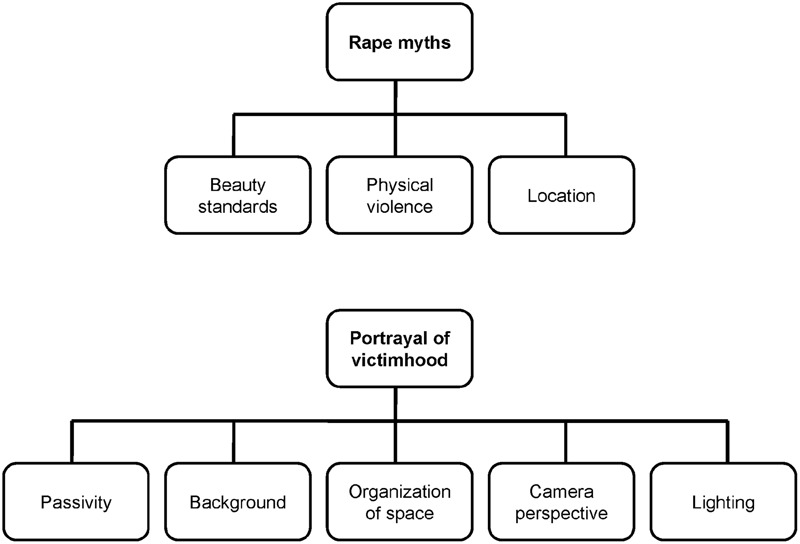
**Themes identified in the data analysis**.

From the dataset, we identified two overarching themes, consisting of respectively three and five sub-themes (see **Figure 1**).

The first theme, “rape myths,” consisted of three sub-themes: “beauty standards,” “physical violence” and “location.” In the second theme, “portrayals of victimhood,” the coders identified the sub-themes “passivity,” “background,” “organization of space,” “camera perspective,” and “lighting.”

The scenes in the pictures portraying a woman were further classified into three categories, independent of the aforementioned (sub-)themes:

(1) Showing a scene heavily implying that an assault is imminent (pre-assault);
(2) Depicting an act of clear physical and/or sexual assault;
(3) Picturing the aftermath of sexual violence (post-assault).

### Theme 1. Rape Myths: Results and Discussion

Rape myths are defined as stereotypical beliefs about sexual aggression that express an exoneration of the perpetrators and blaming of the victims. We identified several of these myths as sub-themes in our visual thematic analysis, hence supporting our first hypothesis.

#### Beauty Standards

This theme occurs in almost all images of this sample that portray women. The women in the photographs fit a typical western norm of female beauty: they are thin, Caucasian, young (approximately early 20s to early 30s) and, as far as the photographs show, able-bodied. Most of them have long hair (blonde or brown) and, if their faces are visible in the picture, they are wearing make-up and jewelry, such as earrings. In the majority of pictures, however, the women’s faces were covered by their hands or arms.

These findings are in line with existing research in this area. While several studies (Len-Ríos et al., 2005; Stanley, 2012) showed in their content analysis of images in newspapers that the majority of the photographs depicted men, if women are present in an image, they are likely to fit existing beauty standards. Research on female beauty standards and objectification has shown that especially magazines marketed toward women fail to present a diverse range of body types and excessively promote thinness as the desired ideal for women (Kilbourne, 1994; Malkin et al., 1999). While this one-sided portrayal of women in terms of body type is common and certainly problematic in advertising, seeing it in the context of reports on sexual violence has other severe implications. This biased visual representation of women feeds into the rape myth that only young women, who fit a western idea of beauty, become victims of sexual violence (Gordon and Riger, 1991). This is a dangerous misconception, as survivors, as well as criminal justice personnel, might not classify an incident as sexual assault if the person concerned does not fit this stereotype. Therefore, they may not report the incident to the police or might face disbelief if they choose to disclose the incident to others.

#### Physical Violence

If an (alleged) perpetrator was present alongside a woman in the photograph (*n* = 15), physical violence against women was either directly shown or heavily implied in all of these pictures. The implication of physical violence was portrayed by the presence of a raised fist or hand aimed at the woman. Other photographs also portrayed the aftermath of a physical attack, showing, for example, women with visible bruising on their faces.

**FIGURE 2:**
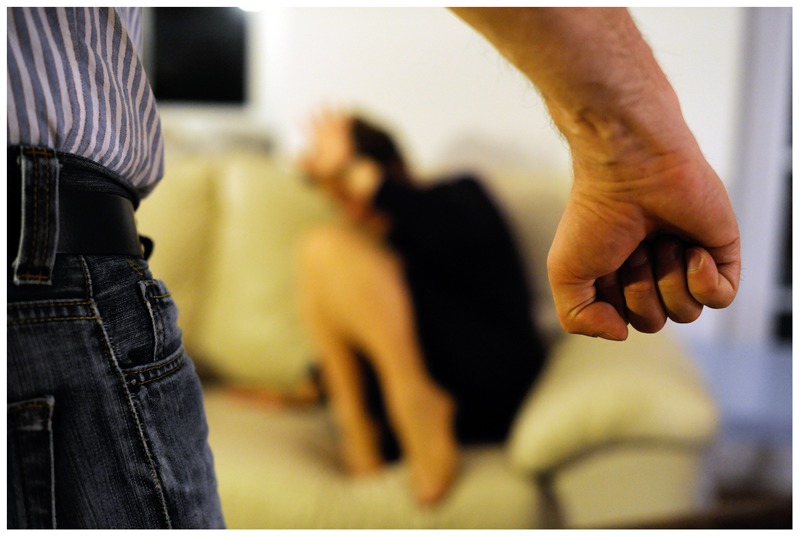
**Sample picture for physical violence (© DPA 2015. Reprinted by permission)**.

Some of the images were very explicit. For example, in one photograph, the hand of an attacker is shown to pull down a woman’s top, exposing her bare chest. One common finding in this sub-theme was the representation of implied physical violence by the perpetrator’s hand. In several pictures, the image of a threatening fist being raised against a woman was present. In others, a disembodied hand was shown, reaching for the victim in a threatening manner (see **Figure 2**).

As stated above, the majority of the images portrayed an act of implied or completed physical violence against women. This is problematic, as it is likely to perpetuate the rape myth that it is only a “real” sexual assault if physical force was used. In a study, Langhinrichsen-Rohling and Monson (1998) presented their participants with different scenarios of marital rape. They found that if the scenario included no prior history of physical abuse, the seriousness of the sexual violence was minimized and participants attributed more blame to the woman than in the scenarios containing physical violence. It is not surprising that journalists looking for a generic image about sexual violence would resort to a photograph portraying (imminent) physical violence. It fits the stereotypical belief most people have about rape as it is frequently portrayed in popular media to be a crime involving physical violence (e.g., Garland et al., 2015). For ethical reasons, an image showing a sexual assault, even if it is a staged one, is likely out of the question. In our sample, there was only one image (described earlier) with a perpetrator forcibly pulling down a woman’s top and exposing her chest, portraying an act of sexual, rather than purely physical violence.

It is interesting that not only online newsletters but also websites providing information for survivors of sexual violence in our sample have used these kinds of images to illustrate their content. This is surprising, as one would expect that these service providers are more sensitive to reproducing misconceptions about sexual violence.

#### Location

The sub-theme “location” described where the scene portrayed in the pictures took place. Those images that showed a “pre-assault” scene almost all depicted a woman walking outdoors on her own. Several of these photos are shot in an alleyway, with the alleged perpetrator watching a woman from behind or from the other side of the road. The majority of the photographs are also shot at night.

Another group of images falling into this sub-theme were images of actual crime scenes in reports on cases of sexual assault. Since the articles belonging to this sub-set of pictures were all about cases of (attempted) sexual assault perpetrated by a stranger, it was not surprising that the photographs showed only “outdoor” crime scenes. In the sample presented here, these were parks, parking garages, and parking lots. In most of these images, the crime scene was shown sealed off by police tape.

The notion that sexual violence is always perpetrated by a stranger who attacks his victim in a remote location is one of the most prevalent rape myths. It is also a stereotypical idea of sexual violence that has long been disregarded as false. In fact, according to Lovett and Kelly (2009) in the majority of cases, the victim knew the perpetrator prior to the assault (48% of cases), whereas only 22% of victims reported that they were sexually assaulted by a stranger. Furthermore, statistics showed that most cases of sexual assault happen in either the victims or perpetrator’s home, rather than in an outdoor location (Lovett and Kelly, 2009).

The idea of “stranger rape” is one of the most popular rape myths, often supported by media reports about rape. The reality, however, is very different. According to an official crime statistic for Germany, only 23% of all reported rape cases in 2015 were committed by a stranger (Bundeskriminalamt, 2015). These findings are a sharp contrast to prevalent views of rape in society. This particular rape myth was also present in the photographs portraying women. All the “pre-assault” pictures showed a woman walking outside, in a remote area. In some of them, the women were being followed by a man. From the composition of the pictures, it can be assumed to supposedly be a stranger. By choosing these kinds of images for their articles, news outlets further perpetuate this already widespread false belief about where sexual assaults happen and who the perpetrators are.

### Theme 2. Portrayals of Victimhood: Results and Discussion

This theme refers to the way that the images discussed here portrayed victimhood in the case of sexual assault.

#### Passivity

**FIGURE 3:**
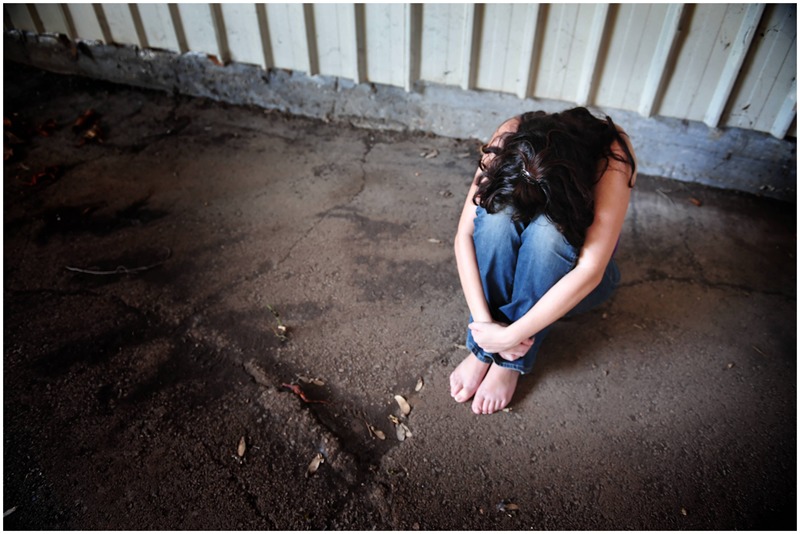
**Sample picture for passivity (© DPA 2015. Reprinted by permission)**.

The sub-theme we identified for this category was “passivity.” It occurred in all three scene categories (pre-assault, ongoing, and post-assault). In the photographs portraying an imminent assault with the perpetrator looming in a threatening manner, the victims were all shown in a cowering position, either on the floor or on a sofa. One distinct similarity in all the photographs was that the women’s faces were only partially or not at all visible. They were covering their faces with their hands, arms, or—in some cases—their long hair. The same was true for the “post assault” images. The vast majority of them showed women cowering in either a home setting or outdoors, with their faces not visible to the observer. In most of these pictures, the women had their arms wrapped around themselves, their legs drawn up against their chest, with their forehead resting on their knees. Another noticeable detail about these images was that some of the women had bare feet, especially the ones set outdoors (see **Figure 3**).

Regarding the fact that in the majority of the cases, the women’s faces were covered by their hands, arms, etc., one might argue that this portrayal is supposed to protect their identity. However, as these photographs were staged as opposed to being snapshots from a real life situation, this explanation is unlikely to hold. While it might not be intuitively related to passivity, another important finding for this sub-theme was the lack of other people present in these images. None of the photographs of women analyzed for this study showed another person in any of the scenes, apart from the victim and the perpetrator. In the pictures where only a woman was present, this gives a distinct impression of the loneliness of her situation. Furthermore, there was no portrayal of a woman seeking any kind of support from a third party after the assault. A more positive and empowering portrayal might have been, for example, a woman disclosing her experience to a female friend or even seeking professional help in a rape crisis center. A vast body of research on consequences of sexual violence on women has shown that counseling is of vital importance for survivors, as it helps them deal with the psychological consequences of the trauma (Mein et al., 2003; Wilken and Welch, 2003). This kind of alternative portrayal would open up a wider representation in terms of options for survivors of sexual violence.

Another noticeable finding was that over all three categories, the women’s body language, as well as their facial expressions (if visible), showed severe distress. This was especially true for the photographs in the “post assault” category. It can be assumed that by choosing these kinds of photographs, the articles’ authors aim to present the detrimental effects of sexual violence on the survivors. While it is important to stress the severe negative physiological and possibly physical consequences of sexual violence, the usage of these kinds of images might not be the best way to do so.

Although the analysis presented here is looking at the portrayal of victimhood in photographs, some of the literature looking at terminology might be applicable in our context. Several studies (e.g., Thompson, 2000; Papendick and Bohner, 2017) found that labels used to refer to women who had been affected by sexual violence carry distinctly different connotations. While the “survivor” label was strongly associated with strength and recovery, the “victim” label was linked with powerlessness, weakness and vulnerability. The latter description linked to the term “victim,” seems to be the one most fitting for the photographs in our analysis. Furthermore, Hockett et al. (2014) argued that “a ‘rape victim’-focused perspective may contribute to a social power hierarchy in which there exist barriers to women’s abilities to construct empowering self-conceptualizations.” This should be taken into consideration when choosing photographs to portray the consequences of sexual violence against women.

#### Background

One aspect the majority of pictures have in common is a non-descriptive background. The photographs show the women in front of what often is a white wall, either in an inside or outside location. Therefore, the images give very little to no context to the viewer about the portrayed situation. Adding to this perceived anonymity and non-descriptiveness is the fact that most of the images do not show the faces of the people portrayed. This makes it difficult to perceive the portrayed people as individuals and could therefore lead the perceivers to emotionally distance themselves from the portrayed situation, taking away empathy for the victim.

#### Organization of Space

In terms of organization of space, it is notable that in the majority of photographs where a *perpetrator is present*, the women are staged in the background, whereas the men are staged in the foreground. The women are presented in the center of the pictures with men to either side of them, only partially shown. At the same time, the amount of space in the pictures dedicated to the women is significantly smaller than that of the male perpetrators.

In several of the pictures where *no perpetrator* is present, the women only take up around a third of the picture, the rest being background (e.g., concrete floor and white wall). If the victims are shown walking, they are staged to one side of the pictures, again taking up relatively little space within the images.

In general, women are socialized to take up little physical space in public when compared to men (Löw, 2016). The same seems to apply to the analyzed pictures. This gives the impression that the women are not the main protagonists in the images.

#### Camera Perspective

Another factor to take into consideration is the camera perspective. As stated above, the women tend to be staged in the background of the images if a perpetrator is present in the pictures. Furthermore, while the women are facing the camera (though often with their faces covered) the perpetrators’ backs are turned toward the viewer. This gives the impression of “looking over the perpetrator’s shoulder” at the woman. In the images where no perpetrator is present, the camera angle is often staged from above, making the viewer look down onto the woman. Taking into account the already passive body language of the women, this adds to the perception of a kind of power from the viewer over the victim.

Additionally, for at least two of the photographs, it is apparent that they cater to what Mulvey (1989) called “the male gaze.” Two of the images show a woman being attacked, her upper body bared. It gives the impression of clearly anticipating a male audience watching as a woman is sexualized as well as assaulted in the image. In his work, Schroeder (1998) explains the connection between the male gaze and an unequal power relationship as follows: “To gaze implies more than to look at – it signifies a psychological relationship of power, in which the gazer is superior to the object of the gaze.”

The fact that most of the images were made to look like they were taken without the women’s knowledge or consent gives the impression of stalkers following their victims. A whole body of literature has addressed the topic of “the male gaze,” mostly in relation to Fredrickson and Roberts’s (1997) theory on objectification. Especially the pictures in the “pre-assault” category could be classified as representing sexual objectification to a certain degree. In all photographs in this category, the picture is taken from behind the woman, often with only her legs or lower body visible, or her head cut off and not part of the picture. Fredrickson and Roberts (1997, p. 174) describe sexual objectification as “the experience of being treated as a body [...] valued predominantly for its use to [...] others.” This is precisely what the photographs in our data set represent. A woman walking alone at night in a public space, portrayed as an object for a man to use—in this case in the context of sexual assault.

In the pictures that portray an ongoing or imminent assault, the framing of the images convey to the viewer that the men in the pictures are the active subjects, whereas the women are seen as the passive objects, being acted upon. Loughnan et al. (2010) found that objectification influences judgments of personhood, specifically the attribution of moral status and mind. Their results suggested that objectification of women leads to them being denied both aspects of personhood. Furthermore, Dworkin (1985) noted that female objectification is an important factor leading to sexual violence against women. Taking all this into consideration, using pictures that objectify women to a certain degree, especially in the context of sexual violence, makes the photographs part of the problem they are trying to raise awareness for.

#### Lighting

**FIGURE 4:**
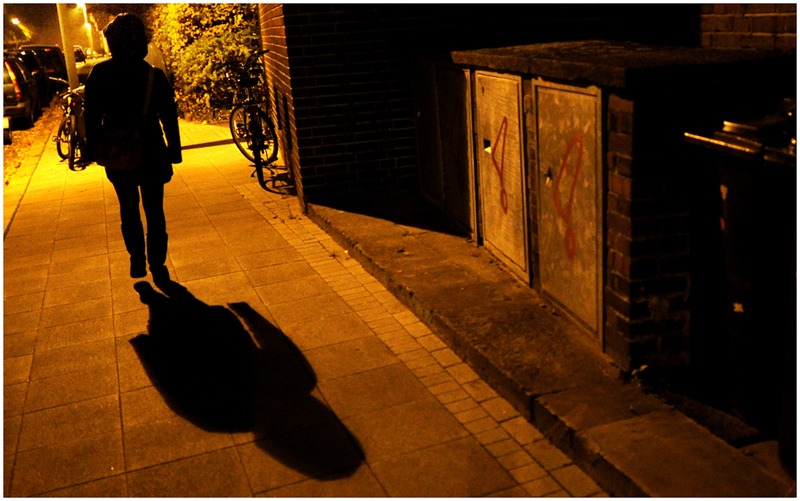
**Sample picture for gaze (© DPA 2015. Reprinted by permission)**.

In general, light is often used in the image as a tool to set the mood. For example, instead of showing the perpetrator himself, one can only see the shadow of his hand on the wall. Additionally, several of the pictures are staged in a dark environment (e.g., after nightfall). All of this could give the impression of a perpetrator “lurking in the shadows” (see **Figure 4**). This adds to the feeling of vulnerability regarding the depicted women.

## General Discussion

In our study, we have investigated visual representations of sexual violence in online news media. Results show that both, sexual violence itself as well as its female victims, are represented in highly stereotyped ways. While several different rape myths are conveyed by the images (e.g., the perpetrator is always a stranger), women are portrayed in a way that feeds into the stereotype of female weakness and vulnerability.

As our analysis has shown, a prominent theme in the photographs was physical violence, either imminent, on-going, or its aftermath. One motivation for news outlets to select potentially upsetting photos for their articles might be that these kinds of images often serve as a way to appeal to the readers’ curiosity. In their study on imagery effects on selective reading, Knobloch et al. (2003) found that incorporating a threatening image into a news article led to more frequent selection of this article and also fostered longer reading times of the affiliated text.

As discussed earlier, several studies have shown that the majority of newspaper images depict women who fit the western beauty standard (Len-Ríos et al., 2005; Stanley, 2012). This appears to be a solid result, independent of the pictures or articles’ context.

While an idealized and limited portrayal of women that severely lacks diversity is not a finding unique to the study presented here, the implications might differ. If women who have been victims of sexual violence do not see themselves presented in the narrow picture the newspapers’ choices of photographs paint, the readiness to report the incident to the police as well as other peoples’ willingness to believe them could be diminished. To provide a more diverse and therefore realistic representation of women as a group, news media outlets should broaden their scope in terms of image choices. Including pictures of, for example, older women, women of color and women with disabilities would tackle the issue of creating awareness that not only young, able-bodied and Caucasian women are victims of sexual violence.

Another important finding from our data was that not only online newspapers resort to these stereotypical photographs for their articles. In our sample, we had eight sources that fall under the category of “providing information and support for victims of sexual violence.” These were, for example, a website from a German police force aiming at crime prevention, a well-known German organization that works in advocating victims’ rights, as well as government websites also working toward crime prevention. Fahmy and Wanta (2007) conducted a survey amongst photo journalists and press photo editors, inquiring about the perceived impact of their work. Results showed that visual journalists believe that their work has a great impact on public opinion. Taking this into account, it is likely that journalists are not aware of the potentially negative impact their choice of image might have. However, organizations specifically tackling issues surrounding crime prevention as well as those working with survivors of sexual violence should be more sensitive about perpetuating false and dangerous stereotypes via their choice of imagery. Overall, future research should focus on investigating differences in how various kinds of news outlets portray the topic of sexual violence, both in writing and regarding the usage of images.

It could be interesting to broaden this research and look at printed newspapers to find out if results can be replicated there. Furthermore, we should analyze media outlets outside of Germany to see if the findings are representative of more than just one country. However, it is likely that our findings are also applicable to other Western countries, since we were able to replicate findings from text-based studies conducted in the United States (Franiuk et al., 2008a,b). Further studies should investigate our assumption that a portrayal of women as powerless and passive is associated with the label of a sexual assault “victim” rather than a “survivor.” If this is the case, it could have implications for the possibility of merging textual and visual findings on this topic.

As we have been able to show in this study that images in news articles perpetuate stereotypes about sexual violence against women, a next step would be to examine if exposure to these images would influence people’s RMA. Since Franiuk et al. (2008a) found this effect for their text-based study, it would be interesting to find out if it holds for visual information as well.

Press photographs play an important role in shaping perceptions regarding social issues and the formation of opinions about reality (Fahmy and Wanta, 2007). It is therefore important to create awareness about the perpetuation of rape myths and about the one-sided portrayal of sexual violence survivors, along with its potentially negative consequences.

## Author Contributions

The author confirms being the sole contributor of this work and approved it for publication.

## Conflict of Interest Statement

The author declares that the research was conducted in the absence of any commercial or financial relationships that could be construed as a potential conflict of interest.
